# Interfering Effects of *In Vitro* Fertilization and Vitrification on Expression of *Gtl2* and *Dlk1* in Mouse Blastocysts 

**DOI:** 10.22074/ijfs.2020.5984

**Published:** 2020-07-15

**Authors:** Elham Movahed, Ronak Shabani, Sara Hosseini, Solmaz Shahidi, Mohammad Salehi

**Affiliations:** 1 Department of Anatomical Sciences, Faculty of Medicine, Iran University of Medical Sciences, Tehran, Iran; 2 Cellular and Molecular Research Center, Iran University of Medical Sciences, Tehran, Iran; 3 Cellular and Molecular Biology Research Center, Shahid Beheshti University of Medical Sciences, Tehran, Iran; 4 Department of Biotechnology, School of Advanced Technologies in Medicine, Shahid Beheshti University of Medical Sciences, Tehran, Iran

**Keywords:** IVF, Mouse, Preimplantation Embryo, Vitrification

## Abstract

**Background:**

Embryo vitrification is a key instrument in assisted reproductive technologies (ARTs). However, there is increasing concern that vitrification adversely affects embryo development. This study intends to assess the effect of vitrification on developmental competence, in addition to expressions of long non-coding RNA (lncRNA) gene trap locus 2 (*Gtl2*) and its reciprocal imprinted gene delta-like homolog 1 (*Dlk1*), in mouse blastocysts.

**Materials and Methods:**

In this experimental study, we have designed three experimental groups: control (fresh blas- tocysts collected from superovulated mice), *in vitro* fertilization (IVF; blastocysts derived from IVF) and vitrification (IVF derived blastocysts subjected to vitrification/warming at the 2-cell stage). Quantitative reverse transcription pol- ymerase chain reaction (qRT-PCR) was performed to assess the expression levels of *Gtl2* and *Dlk1* in the blastocysts.

**Results:**

The results showed that vitrification group had significantly lower blastocyst and hatching rates compared to the IVF group (P<0.037) and (P<0.041), respectively. Gtl2 was down-regulated and Dlk1 was up-regulated following the IVF and vitrification (P<0.05).

**Conclusion:**

These results suggested that IVF and vitrification disturbed genomic imprinting and lncRNA gene expres- sions, which might affect the health of IVF children.

## Introduction

Embryo cryopreservation, an important component of assisted reproductive technologies (ARTs), has considerably improved the clinical results of this technology (1). Vitrification and slow freezing are two routine methods for embryo cryopreservation. Vitrification is routinely used in ART clinics because of its higher survival rate post-warming, in addition to its simple and inexpensive technique in comparison with slow freezing. However, it is still not known whether vitrification affects the health of adults who were conceived by ART, with respect to the cytotoxicity of high concentrations of cryoprotectants used for vitrification and stresses from high cooling and warming rates (2).

Long non-coding RNAs (lncRNAs) are transcripts with more than 200 up to several thousand nucleotides. Although most of these molecules do not have protein coding capacity, some of them code small peptides of less than 100 aminoacids (3). It is anticipated that thousands of lncRNAs exist in the mammalian transcriptome and, until now, nearly 15000 human lncRNAs have been characterized (4, 5).

lncRNAs have important regulatory roles in many cellular processes such as gene expression, imprinting, cytoplasmic scaffolds and intracellular trafficking. They affect cell function during development and differentiation (4, 6). In addition, correlated with the expression of pluripotency markers, lncRNAs play role in the embryonic stem cell regulatory (5). lncRNAs are involved in the numerous pathological conditions, including oncogenesis (4). On the other hand, a regulatory epigenetic mechanism (genomic imprinting) causes asymmetric parental allele expressions in a series of mammalian genes (7, 8). In imprinting genes, one of the parental alleles is expressed whereas another allele is methylated and silenced. Disruption in genomic imprinting results in pathological conditions such as Beckwith-Wiedemann Syndrome (BWS) and Angelman Syndrome (AS) (8). Gene trap locus 2 (*Gtl2*; approved symbol: *Meg3*) and delta-like homolog 1 (*Dlk1*) are reciprocally imprinted gene located on mouse distal chromosome 12. *Gtl2* is a chromatin-interacting lncRNA expressed from the maternal allele, whereas *Dlk1* is a paternally expressed gene. The *Dlk1/Gtl2* imprinting locus has an momentous role in embryonic development and growth (9). Previous researches have established that epigenetic disruption of this imprinted locus is related to facial dysmorphisms, skeletal abnormalities and muscular hypertrophy. Additionally, loss of imprinting in *DLK1/GTL2* has been reported in pheochromocytoma, neuroblastoma and Wilms’ tumour (10-12).

A review of the literature showed no data that pertained to an association between embryo vitrification and lncRNA expressions. Thus, considering the importance of *Dlk1* and *Gtl2* in embryo development, we sought to investigate their expressions *in vitro* fertilization (IVF) pre-implanted embryos, embryos subjected to vitrification and warming, and fresh blastocysts. Here, we made use of a mouse embryo model because of the ethical issues that pertain to research on human embryos.

## Materials and Methods

This experimental study, approved by and Ethical Committee of Shahid Beheshti University of Medical Sciences (Tehran, Iran, Ethical permission number: IR.SBMU. RETECH.REC.1396.997). All animal experiments were conducted in compliance with the guidelines established by this university for the keeping and manipulate of laboratory animals.

### Materials

All chemicals and reagents were obtained from Sigma Chemical Company (St. Louis, USA) unless otherwise noted.

### Animals

We obtained 6-8 weeks old female and 10-weeks old male NMRI mice from Royan Institute (Tehran, Iran) to use in this study. The mice were accommodated under the controlled conditions of 12 hours light: 12 hours dark photoperiod at room temperature (22 ± 2°C) and 50 ± 10% humidity with ad libitum use of food and water. The animals were killed by cervical dislocation.

### Experimental design

**Fig 1:**
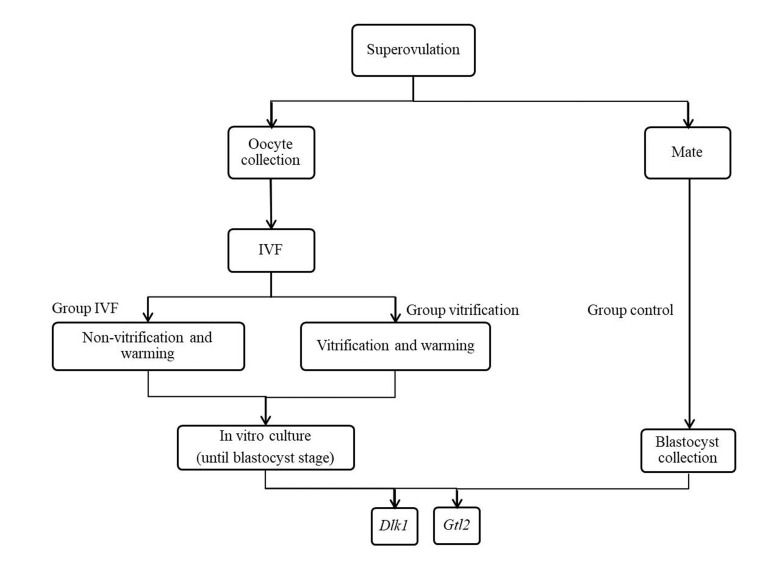
Experimental design and IVF; *In vitro* fertilization, Dlk1; Delta-like homolog 1, and Gtl2; Gene trap locus 2.

Female mice were superovulated by intraperitoneal (IP) injection of 10 IU pregnant mare serum gonadotropin (PMSG; Pregnecol®, Australia), followed 48 hours later by 10 IU human chorionic gonadotropin (hCG; Pregnyl). The experiment was carried out on three treatment groups: control, IVF, and vitrification as shown in Figure 1.

In the control group, after hCG injection, female mice were mated with male mice. Successful mating was verified by the detection of a vaginal plug, the next day morning. Fresh blastocysts were collected from the mice uteri by flushing the uterine horns with FHM flushing media 94 hours posthCG, according to the previous study (13). The blastocysts were used for RNA extraction and reverse transcription.

In the IVF and vitrification groups, we collected the cumulus oocyte complexes containing metaphase II (MII) oocytes from the oviduct ampullae 14-16 hours after hCG injection. The oocytes were released into FHM medium and then transferred to 50 μl droplets of human tubal fluid medium (HTF) supplemented with 4 mg/ml bovine serum albumin (BSA).

### *In vitro* fertilization

IVF was performed as formerly explained (14). Sperms were collected from the male mice. The cauda epididymides and vas deferens were isolated and placed in a petri dish containing previously equilibrated HTF medium (37°C, 5% CO2 in air). The sperms were passively released into the culture by using pointed forceps and a razor blade. The suspended sperms were incubated at 37°C for 45 minutes to allow capacitation. Capacitated motile spermatozoa were added to 50 μl IVF drops to reach 1 × 10^6^ sperm/ml concentrations. Subsequently, they were incubated in a humidified atmosphere of 5% CO_2_ in air at 37°C under mineral oil for 5-6 hours. Next, the in vitro-derived zygotes were washed in FHM medium and cultured in potassium simplex optimized medium (KSOM) supplemented with 4% BSA under the same conditions to allow for further development. After 24 hours, we divided the 2-cell embryos into two groups. In the IVF group, the embryos were maintained in KSOM for 72 hours until the blastocyst stage. In the vitrification group, the 2-cell embryos were vitrified/warmed and then cultured under the same conditions as the IVF group, for 72 hours, to reach the blastocyst stage. Finally, the rates of development at the 4-cell, 8-cell, morula and blastocyst stages were assessed in both groups. The blastocysts were used for RNA extraction and reverse transcription.

### Vitrification and warming

In the vitrification group, the 2-cell embryos were vitrified by the cryotop method with Kitazato Vitrification Kit (Kitazato Biopharmaceuticals, Japan), as previously described (15). Briefly, embryos were equilibrated in equilibration solution (ES) with 7.5% (v/v) ethylene glycol (EG) and 7.5% (v/v) dimethyl sulfoxide (DMSO). After 3 minutes, the embryos were exposed to the vitrification solution (VS) containing 15% (v/v) EG, 15% (v/v) DMSO and 0.5 mol/l sucrose for less than 1 minute. Next, 3-5 embryos with minimal VS were loaded onto the inner surface of the cryotop and immediately submerged in liquid nitrogen (LN2), followed by capping and storing in LN2 for up to 2 weeks. Vitrification processes were carried out at room temperature. For warming, the embryos were exposed to decrease concentrations of sucrose on a 37°C hot plate, as follows: 0.5 M sucrose for 1 minute, 0.25 M sucrose for 3 minute and 0.125 M sucrose for 5 minute. Finally, the embryos were placed for 3 minute in a 0 M washing solution and they were assessed for survival by observing the intactness of zona pellucida and blastomeres. The surviving 2-cell embryos were cultured in KSOM medium in an incubator at 37°C and 6% CO_2_ to allow further development to the blastocyst stage. All media used for warming were incubated at 37°C for 30 minutes before warming.

### RNA extraction and complementary DNA synthesis

RNA extraction, complementary DNA (cDNA) synthesis, and quantitative reverse-transcription PCR (qRTPCR) analysis were carried out according to the previous study protocols (16). Briefly, two blastocysts in each replicate of each experiment were pipetted into microtubes containing 1.5 μl lysis buffer. We added 5 μl nucleasefree water and 2 μl random hexamer to each sample and then placed the samples in a BioRad thermocycler for 5 minutes at 75°C. Immediately afterwards, the microtubes that contained the reaction product were placed on ice, followed by the addition of 5x RT buffer, 200 u RT enzyme, 10 mM dNTP, and 10 U RNase inhibitor to each reaction for cDNA synthesis. Reverse transcription (RT) reaction was performed in the thermocycler with the following amplification program: 25°C for 10 minutes, 37°C for 15 minutes, 42°C for 45 minutes and 72°C for 10 minutes. The samples were left at 4°C overnight. PCR mixture, consisted of 5 μl Master Mix (Taq DNA Polymerase Mix Red-MgCl; Amplicon, Denmark), 3 μl nuclease-free water, 1 μl cDNA, and 1 μl specific primer (Table 1) was added to each PCR microtube to amplify cDNA product. The endogenous control (β2m) and the investigated genes were amplified according to the following PCR cycle: 94°C for 3 minues (denaturation), 94°C for 30 seconds (denaturation), 60°Cfor 45 seconds (annealing) and 72°C for 45 seconds (extension), followed by 40 cycles. A final elongation step was carried out at 72°C for 10 minutes. The amplification products were loaded and run alongside a DNA ladder on a 2% agarose gel in TAE and, after 25 minutes, they were observed under short-wave UV.

### Quantitative reverse transcription PCR (qRT-PCR) analysis

qRT-PCR was executed to evaluate the amount of *Dlk1* and *Gtl2* expressions by using a Rotor Gene Q instrument (Qiagen, USA). Table 1 lists the primer sequences applied for qRT-PCR. qRT-PCR reaction were conducted in a total volume of 13 μl reaction containing 1 μM of each primer for the indicated genes and 1 μM of the synthesized cDNA based on the manual for the DNA Master SYBR Green 1 mix (Roche Applied Sciences, Germany). Cycling program for the RT-PCR was as follows: 2 minutes at 95°C, and 40 cycles of 5 seconds at 95°C, 30 seconds at 60°C, 10 seconds at 72°C. Melting curve examination for all amplification reactions confirmed the particular amplification peaks and lack of primer-dimer formation. *β2m* was the endogenous internal house-keeping gene for RT-PCR data normalization. We used the Relative Expression Software Tool (REST, version 2009) for qRTPCR data analysis.

### Statistical analysis

Statistical analyses were performed by applying the Statistical Package for the Social Science software, version 16 (SPSS, USA). Cleavage and developmental ratio to blastocysts stage between IVF and vitrification groups were compared by the non-parametric Mann- Whitney test. The relative gene expression levels of *Gtl2* and *Dlk1* were analyzed by REST software (Qiagen). P<0.05 was regarded as statistically significant.

## Results

### Embryo development

We assessed the effect of vitrification on developmental competence of preimplantation embryos. The 2-cell embryos obtained from IVF in three runs were divided into two groups. Totally, for the IVF group, there were 170 cultured 2-cell embryos. In the vitrification group, 166 embryos (2-cell) were vitrified/ thawed. The vitrification group had a survival rate of 96.72% ± 2.93, after vitrification and warming. We compared the percentage rates of the 4-cell, 8-cell and morula stages between the IVF and vitrification groups. There was no significant difference between these two groups, in terms of cleavage rate. The blastocyst (64.04% ± 10.16) and hatching (48.51% ± 10.92) rates in the vitrification group were significantly lower than the blastocyst (82.63% ± 2.56; P<0.037) and hatching (69.22% ± 5.20; P<0.041) rates in the IVF group (Table 2).

**Table 1:** Details of primers applied for RT-PCR and qRT-PCR


Genes	Nucleotide sequences (5′–3′)	Tempreture (°C)	GC%	Self-complementarity	Accession number

*Gtl2*	F: CTGAAGAAAAGAAGACTGAGGAC	56.8355.86	43.4850.00	3.003.00	NR_003633.3
	R: CGATTTACAGTTGGAGGGTC				
*Dlk1*	F: CTGCGAAATAGACGTTCGG	56.5657.14	52.6357.89	4.004.00	XM_006515457.3
	R: GTACTGGCCTTTCTCCAGG				
*β2m*	F: AGACTGATACATACGCCTGC	57.2056.80	50.0050.00	3.006.00	M_009735.3
	R: ATCACATGTCTCGATCCCAG				


RT-PCR; Reverse transcriptio polymerase chain reaction, and qRT-PCR; Quantitative reverse transcription polymerase chain reaction. GC; Guanine - Cytosine Percent.

**Table 2:** Development of 2-cell mouse embryos in vitro fertilization and vitrification groups


Group	2-cell embryos(n)	Survival rate	4-cell rate	8-cell rate	Morula rate	Blastocysts rate	Hatched rate

IVF	177	100%(170/170)	95.36% ± 1.17(162/170)	92.19% ± 2.83(157/170)	88.04% ± 2.59(150/170)	82.63% ± 2.56^*^(141/170)	69.22% ± 5.20^**^(117/170)
Vitrification	166	96.72% ± 2.93(160/166)	92.32% ± 2.64(148/160)	84.49% ± 6.92(135/160)	76.6% ± 7.58(123/160)	64.04% ± 10.16^*^(102/160)	48.51% ± 10.92^**^(77/160)


Data are presented as mean ± SD or n (%). *Significant difference (P<0.037), **Significant differences (P<0.041)

### *Dlk1* and *Gtl2* expression levels

**Fig 2:**
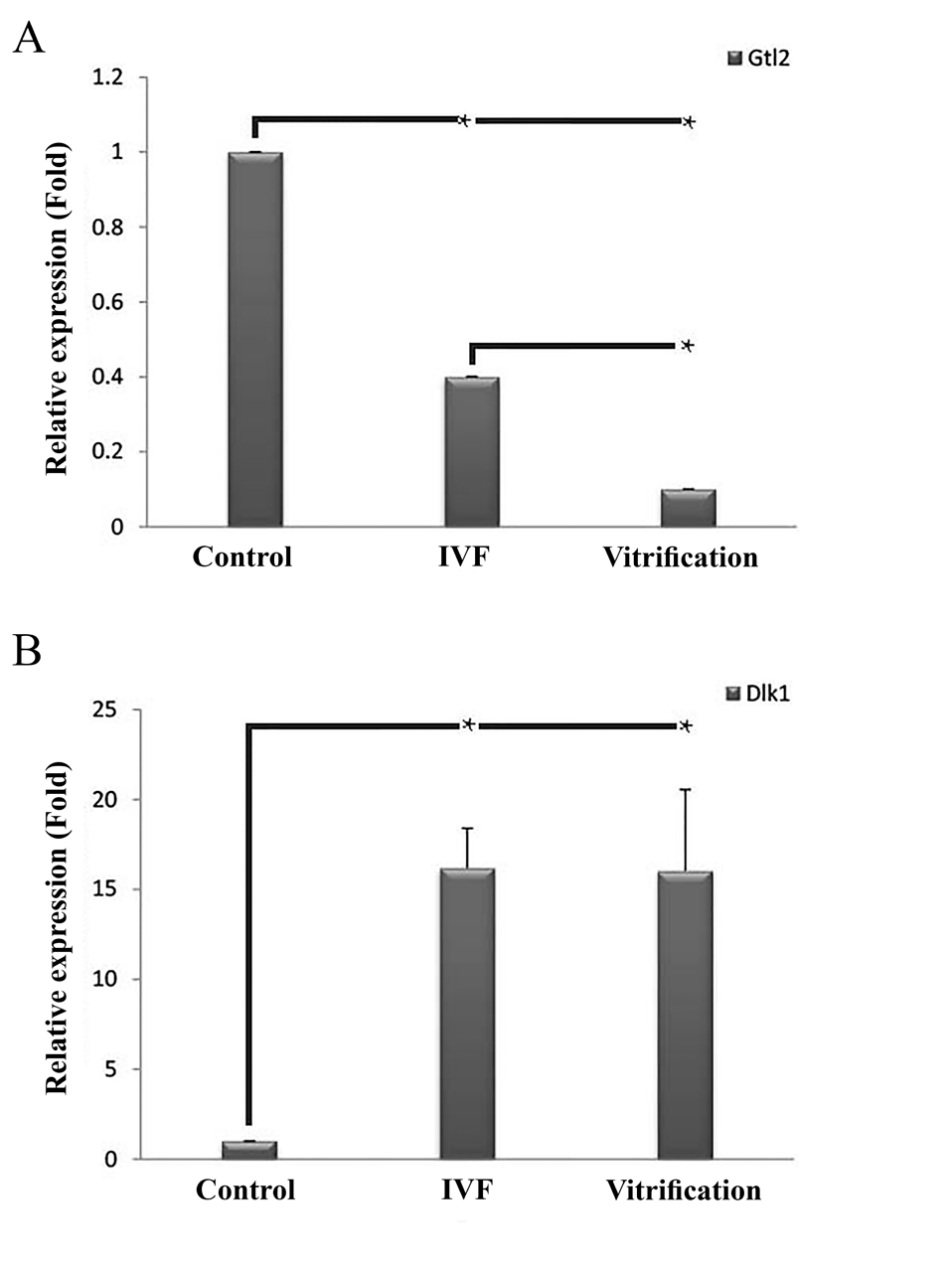
Relative expression levels of mouse of gene trap locus 2 (*Gtl2*) and delta-like homolog 1 (*Dlk1*) in the blastocysts of the experimental groups. **A.** The expression levels of Gtl2 and **B.** *Dlk1*, *; P<0.05.

qRT-PCR was implemented to appraise the expression levels of the lncRNA *Gtl2* and *Dlk1* gene in blastocysts. Gtl2 expression was down-regulated in the IVF and vitrification groups compared to the control group. *Gtl2* was less expressed in the vitrification group compared to the IVF group (P<0.05, Fig . 2A). *Dlk1* was up-regulated in the IVF and vitrification groups compared to the control group (P<0.05). There was no difference between the IVF and vitrification groups, in terms of *Dlk1* expression (P<0.05, Fig .2B).

## Discussion

Vitrification is an encouraging technology to cryopreserve gametes and embryos in ART clinics. The main challenge faced by researchers is to evaluate the consequences of this process on healthy and affectedadults conceived by IVF and optimization of this important technology (2, 17, 18). In this study, we assessed the influence of vitrification using cryotops on developmental competence and expression levels of the lncRNA *Gtl2* and *Dlk1* gene in pre-implanted mouse embryos.

We assessed the embryonic developmental potential after vitrification by comparing cleavage, blastocysts and hatching rates of the non-vitrified embryos (IVF group) compared to the vitrified embryos (vitrification group). The results showed that vitrification/warming at the 2-cell stage significantly decreased blastocysts and hatching rates in mouse preimplantation embryos. This finding provided evidence of the adverse effects of vitrification on development of preimplantation embryos. This result supported earlier observations where vitrification negatively impacted development of preimplantation mouse embryos (2, 19, 20). Vitrification generates increased levels of reactive oxygen species (ROS). ROS leads to interrupted cell function and division. Thus, to some extent, high ROS levels are in charge of lower developmental competence in embryos subjected to vitrification (20, 21). Most likely, antioxidant enzymes such as SOD and catalase, which are responsible for cell defense against ROS in normal conditions, are destroyed during vitrification (20). Additionally, it has been shown that vitrification leads to zona hardening of preimplantation embryo. Thus, zona hardening could be the explanation of the decrease in hatching rate subsequent to vitrification. Difficulty in hatching process could have negative effect on implantation potential of embryo (22).

Recent evidence suggests that ART, including superovulation, IVF and vitrification cause a disturbance in genetic and epigenetic mechanisms in the pre-implanted embryo affecting health of the children conceived by ART (2, 17, 18). However, previous studies have not addressed lncRNA changes in embryos derived from ART. lncRNA Gtl2 and its reciprocal imprinted gene, *Dlk1*, are important for normal development of embryo tissues such as the brain and bones, in addition to the postnatalregulation of neural system and metabolism (23). *Gtl2* has also a major anti-tumor activity mediated through p53- dependent and p53-independent pathway in humans (4). Through RNA–DNA triplex structures, *Gtl2* takes part in the regulation of TGF-b signaling pathway genes (24). *Dlk1* codes a transmembrane protein and it is fundamental to normal cellular differentiation. It plays a major role in carcinogenesis. Therefore, the central thesis of this paper is whether IVF and embryo vitrification interfere with the expression of lncRNA Gtl2 and its reciprocal imprinted gene, *Dlk1*, in mouse blastocysts. In the maternal allele, the intergenic differentially methylated region (IG-DMR) of *Dlk1/Gtl2* is unmethylated and there is expression of *Gtl2*. However, in the paternal allele, the IG-DMR of *Dlk1/Gtl2* is methylated, and *Dlk1* is expressed (9). In this study, we observed decreased *Gtl2* expression and increased Dlk1 following IVF and vitrification. Disruption in the imprinting of other imprinted genes following IVF and vitrification have been shown in the previous papers (25, 26).A possible explanation for our result might be decline in level of DNA methylation. Prior studies noted that IVF and vitrification decreased DNA methylation in blastocysts (2, 13, 26). Decreased DNA methylation might be attributed to disturbances in DNA methyltransferases (Dnmts) expressions following IVF and vitrification, as the previous study revealed that IVF and vitrification result in increased relative expression levels of miR-29a and miR-29b and consequently decrease in Dnmt3a and Dnmt3b relative expression levels, as the target genes of *miR-29a* and *miR-29b* and responsible for de novo DNA methylation (13).

## Conclusion

In conclusion, vitrification at the 2-cell stage adversely affected preimplantation mouse embryo development. In addition, IVF and vitrification interrupted the expressions of lncRNA Gtl2 and its reciprocal imprinted gene, Dlk1, in mouse blastocysts. This study was the first to assess expression of lncRNAs following ART manipulation. Due to the importance of lncRNAs in embryo development, more research would be needed to evaluate lncRNA expressions in embryos conceived by ART.
